# Cognitive Impairments in Hashimoto’s Encephalopathy: A Case-Control Study

**DOI:** 10.1371/journal.pone.0055758

**Published:** 2013-02-08

**Authors:** Jianhong Wang, Jun Zhang, Lan Xu, Yunbo Shi, Xunyi Wu, Qihao Guo

**Affiliations:** 1 Department of Neurology, Huashan Hospital, State Key Laboratory of Medical Neurobiology, Shanghai Medical College, Fudan University, Shanghai, China; 2 Department of Radiology, Huashan Hospital, Shanghai Medical College, Fudan University, Shanghai, China; University of California, San Francisco, United States of America

## Abstract

**Background/Aims:**

Hashimoto's encephalopathy is considered as a treatable dementia, but it is often misdiagnosed. We investigated cognitive impairment and the MRI pathology of Hashimoto's encephalopathy patients.

**Methods:**

The study comprised eight patients with Hashimoto's encephalopathy, 16 patients with mild Alzheimer’s disease and 24 healthy subjects. A neuropsychological battery included assessments of memory, language, attention, executive function and visuospatial ability. Cranial MRI was obtained from all Hashimoto's encephalopathy patients.

**Results:**

Hashimoto's encephalopathy and mild Alzheimer’s disease showed cognitive impairments in episodic memory, attention, executive function and visuospatial ability, but naming ability was unaffected in Hashimoto's encephalopathy. The MRI of Hashimoto's encephalopathy showed leukoencephalopathy-like type or limbic encephalitis-like type; the lesions did not affect the temporal cortex which plays a role in naming ability.

**Conclusion:**

Except that the naming ability was retained, the impairments in cognitive functions for the Hashimoto's encephalopathy patients were similar to those of Alzheimer’s disease patients. These results were consistent with the MRI findings.

## Introduction

Hashimoto's encephalopathy (HE) is a rare, controversial neurological disorder associated with high titers of antithyroid antibodies [1], [2]. Patients are mostly women [2]. Typically, HE is a steroid-responsive, relapsing–remitting and progressive encephalopathy. The clinical manifestations of HE are various, but frequently involve disorders of consciousness (e.g., confusion, coma), stroke-like episodes, cognitive decline, seizures (including focal or generalized seizures), psychiatric manifestations (such as depression, mania or hallucinations), myoclonus and movement disorders [3]–[5]. Although cognitive impairment is commonly described in cases of HE, the exact nature of the impairment and the associated neuroimaging pathology remain unclear.

An investigation of the cognitive symptoms and homologous neuroimaging manifestations of HE will contribute to the differential diagnosis of dementia. Furthermore, as the diagnosis criteria of HE is controversial, such research would help to confirm the diagnosis of HE, and contribute to the assessment of responses to treatment [6]. This study aims to analyze the cognitive impairment characteristics of HE by comparing HE patients with early-onset Alzheimer’s disease (AD) patients and a normal control group. In addition, the magnetic resonance imaging (MRI) data of HE patients is retrospectively analyzed to investigate the pathological status of HE.

## Materials and Methods

### Participants

Participants included 8 patients with HE and 16 patients with mild AD, who were recruited from the Memory Clinic, Department of Neurology, Huashan Hospital, from January 2010 to March 2012.

The HE patients (the case group) were carefully selected according to the following criteria: 1) clinical manifestations of HE in accordance with generally accepted diagnostic criteria [2], [7]: cognitive impairment (Clinical Dementia Rating, CDR = 1) with or without neuropsychological symptoms; seizures; focal neurological deficits or movement disorders; 2) elevated titers of antithyroid antibodies in serum, including antithyroid peroxidase antibodies (anti-TPO; normal <60 IU/ml), and antithyroglobulin antibodies (anti-TG; normal <60 IU/ml); 3) negative results for infectious diseases (determined using the Treponema pallidum particle agglutination test and rapid plasma regain test, anti-HIV antibody, anti-rubella IgG, IgM, anti-measles IgM, anti-cytomegalovirus IgG, IgM), tumor antigens (CEA, AFP, CA199, CA125, NSE, SCC et al), para-neoplastic antibodies (immunoblot anti-Hu neuronal nuclear antibody IgG, immunoblot anti-Yo neuronal nuclear antibody IgG, immunoblot anti-Ri neuronal nuclear antibody IgG, immunoblot anti-CV_2_ neuronal nuclear antibody IgG et al), and other immune antibodies including ANA, ENA, ANCA, dsDNA, as well as serum voltage gated potassium channel (VGKC) antibodies and anti-NMDA receptor antibodies; 4) cognitive assessment was performed prior to steroid treatment; 5) to achieve the neuropsychological tests, we excluded the patients with disorders of consciousness, visual or auditory deficits, or obvious symptoms of medical or psychiatric dysfunction (including mania and depression) within the previous month; 6) EEG showed no triphasic wave.

The mild AD patients (patient control group) met the following criteria [8]: 1) diagnosis of AD according to the DSM-IV [9], (CDR = 1); 2) to achieve the neuropsychological tests, we excluded the patients with disorders of consciousness, obvious medical diseases, psychiatric/psychological dysfunction (including anxiety, mania and depression) within the previous month or visual or auditory deficits.

Twenty-four healthy old subjects from urban communities in Shanghai were chosen as the normal control group using cluster sampling. All participants were native Chinese speakers. There were no statistical differences among the three groups in terms of sex or educational level (*P*>0.05; Table 1). Informed written consent was obtained directly from patients or family members. The ethics committee at Huashan Hospital, Fudan University approved the protocol.

**Table 1:** General information for the three groups (means±SD).

Index	HE group(n = 8)	Normal group(n = 24)	mild AD group(n = 16)	F(χ2) value	*P* value
Age	38.88±11.57	60.25±6.64* *	60.44±5.76	28.204	<0.001
Years of Education	12.50±2.45	13.38±2.39	11.38±3.20	2.642	0.082
Gender(M:F)a	5∶3	5∶11	15∶9	4.087	0.130
MMSE Total score	21.63±1.51	28.75±1.19* *	23.31±2.94	56.586	<0.001
CESD	7.58±3.47	9.16±7.99	9.00±5.07	0.197	0.898

CES-D = Center for Epidemiologic Studies Depression Scale.

a = χ2 analysis.

* * = *P*<0.01, normal group vs. HE group.

### Neuropsychological Assessment

Participants were given neuropsychological tests by a trained rater who was unaware of the study aims or the patient diagnosis. A comprehensive neuropsychological battery was used, which included assessments of memory, language, attention, executive function and visuospatial ability. All tests have been shown to have good reliability and validity when used in Chinese populations [10]. The specific tests employed were: the Center for Epidemiologic Studies Depression Scale [11]; the Auditory Verbal Learning Test (AVLT) [12], [13]; the Rey-Osterrieth Complex Figure Test (CFT) [13], the event-based prospective memory test (EBPM) and the time-based prospective memory test (TBPM) [14], [15]; the Boston Naming Test (BNT, 30-item version) [16]; the verbal fluency test (VFT) [17]–[19]; the Trail Making Test, parts A and B (TMT-A, TMT-B) [20]; the Symbol Digit Modalities Test (SDMT) [21]; the Stroop Color-Word Test (SCWT) [22]; the similarity test [23]; the stick test [24]; and the clock drawing test (CDT) [25], [26]. (Please refer to Dementia and Geriatric Cognitive Disorders, 2011; 31∶284–290 for details).

### MRI Examination

All MR images were obtained using a 3.0-T system (Signa VH/i, GE Healthcare, Milwaukee, WI, USA) equipped with a standard head coil. The subject’s head was immobilized in the head coil with foam padding. The conventional MRI sequences included axial T1-weighted imaging (T1WI), T2-weighted imaging (T2WI) and fluid-attenuated inversion recovery (FLAIR). Coronal images were used because the hippocampus is more easily identified in this acquisition plane.

### Statistical Analysis

The ages of subjects were significantly different between the three groups. Therefore, the overall differences between the three groups were assessed using ANCOVA to remove the effect of age. Post-hoc pair-wise comparisons between the groups were made using the least significant differences test. The level of significance (α) was set at 0.05.

## Results

The average age of the HE group was 38.88±11.57 years (3 males, 5 females; Table 1). The educational levels included elementary school (n = 1), junior high school (n = 2), senior high school (n = 3) and college (n = 2). Clinical examination revealed that symptoms/signs of HE included seizures (n = 4), psychological or psychiatric disorders (n = 3), stroke-like episodes (n = 2) and corticospinal abnormalities (n = 1). The clinical features of the HE cases are summarized in Table 2.

**Table 2:** Clinical and laboratory findings for the HE group.

Case	Age (yrs)	Sex	Clinical presentation	Special examinations	Treatment	Evolution
1	58	F	Partial complex seizures, cognitive impairment	Hippocampus swelling and N angio, EEG diffuse slowing, sharp waves>left	A, S	Occasional relapses, then stable
2	47	F	Partial complex and generalizedseizures,cognitive impairment	MRI bilateral medial temporal andhippocampus FLAIR hypersignal, EEG diffuse slowing, sharp waves>right	A, S	Improvement after ttt
3	41	F	Cognitive impairment, apathy	MRI white matter alterations, N EEG	S	Improvement after ttt, persistent mild cognitive impairment
4	24	F	Focal and generalized seizures, cognitive impairment	MRI left medial temporal and amygdalaFLAIR hypersignal, N EEG	A,S	Improvement after ttt, seizure free
5	38	M	Cognitive impairment, anxiety	Hippocampus mild swelling, EEG delta slowing	S,T4	Slight improvement after ttt
6	46	M	Generalized seizures, cognitive impairment	Hippocampus mild swelling, EEG slowing	S	Improvement after ttt, no seizure relapse
7	31	F	cognitive impairment,occasional stupor	MRI white matter alteration, EEGdiffuse slowing,	S	Significant improvement after ttt, relapses
8	26	M	cognitive impairment	Hippocampus well-stacked, N EEG	S	Improvement after ttt

N, normal; angio, cerebral angiography; CT, computed tomography; EEG, electroencephalogram; MRI, magnetic resonance imaging; S, steroid; A, anticonvulsant; t, treatment; T4, levothyroxin.

The tests for antithyroid antibodies (conducted to satisfy the diagnostic criteria) revealed that anti-TPO levels were greater than 1,300 IU/ml in five HE patients; the other three patients had anti-TPO levels greater than 400 IU/ml (i.e., 1253.63 IU/ml, 1113.2 IU/ml and 495.3 IU/ml). Anti-TG levels were greater than 90 IU/ml in all eight cases. Three of the patients were hyperthyroid.

**Figure 1:**
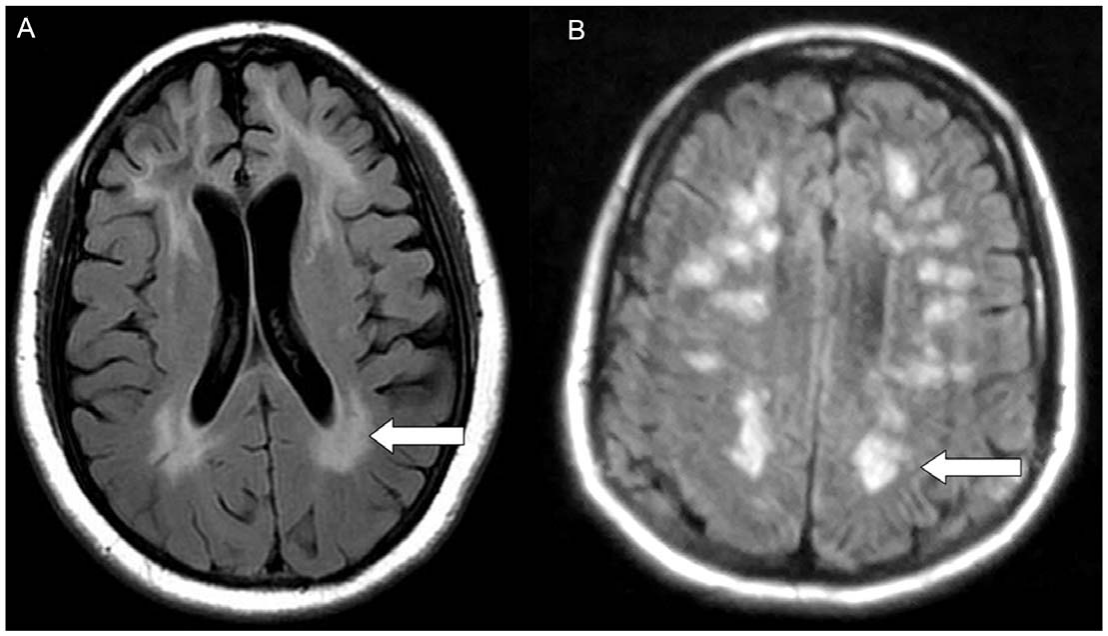
Leukoencephalopathy-like manifestations on MRI images. (A, B)Axial MRI images demonstrate widespread periventricular hyperintense signals in the cerebral white matter.

**Figure 2:**
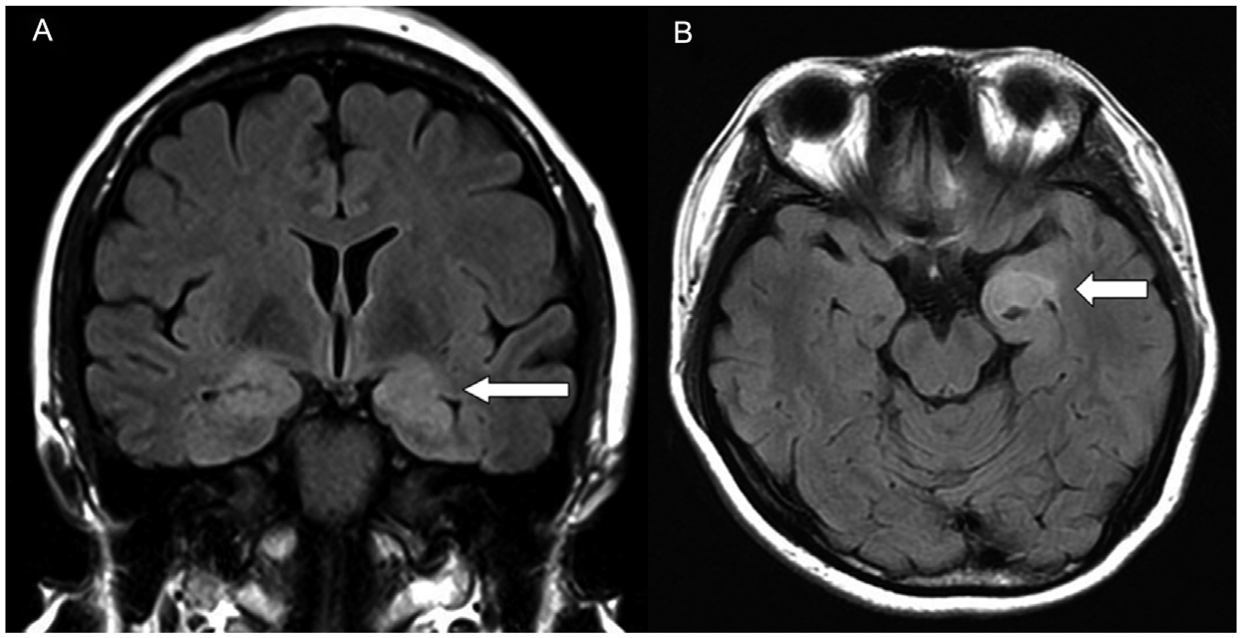
Limbic encephalitis-like manifestations on MRI images. (A) Coronal MRI image shows bilateral medial temporal and hippocampus hypersignal on FLAIR. (B) Axial MRI image shows left medial temporal and amygdala hypersignal on FLAIR.

Cerebral MRI showed that manifestations of HE varied from white matter alterations to hippocampus swelling to medial temporal lobe/hippocampus lesions (Figure 1(A, B), Figure 2(A, B)).

The means ± SD of the neuropsychological test scores are summarized in Table 3. As expected, HE patients had greater cognitive impairment than the control subjects in most tests (*P*<0.05) except EBPM (2; non-language response), TBPM (2), BNT, VFT (fruit), stick test (copy) and CDT. Apart from BNT, there were no significant differences between mild AD and HE patients.

**Table 3:** Comparison of neuropsychological tests among the three groups (mean±SD).

Index	HE group(n = 8)	NC group(n = 24)	mild AD group(n = 16)	F value	*P* value
AVLT1R	1.88±1.36	4.67±1.31a	2.06±1.39	22.097	<0.001
AVLT2R	2.88±1.55	5.29±1.40b	3.50±1.21	9.874	<0.001
AVLT3R	3.38±2.13	6.88±1.80a	3.75±1.13	19.572	<0.001
AVLT4R-short delay recall	1.25±1.75	5.42±1.53a	0.50±0.82	74.039	<0.001
AVLT5R-long delay recall	0.63±0.92	5.25±1.36a	0.19±0.54	112.140	<0.001
AVLT6R-cue recall	0.38±0.74	5.00±1.50a	0.31±0.70	84.918	<0.001
AVLT7R-recognition	13.63±2.56	21.71±1.46a	13.75±3.00	77.264	<0.001
CFT-delay recall	2.75±2.12	14.88±6.48a	3.25±3.72	33.947	<0.001
EBPM-VR	0.38±0.74	3.42±2.60b	0.25±0.58	14.271	<0.001
EBPM-NVR	2.25±1.67	3.83±0.48	2.50±1.59	6.726	0.003
TBPM- VR	0.13±0.35	3.96±2.53a	0.31±0.60	21.290	<0.001
TBPM- NVR	0.50±0.54	2.29±2.01	0.25±0.58	9.565	<0.001
SDMT-accidental memory	0.75±1.17	3.79±2.80a	0.75±1.07	12.716	<0.001
BNT (30 item)	19.38±4.17	22.08±3.40	20.25±4.30	1.252	0.296
VFT-Animal	10.88±2.70	16.13±3.78b	11.00±3.27	11.841	<0.001
VFT-Fruit	7.88±2.53	11.08±2.36	7.06±1.73	16.211	<0.001
VFT-Vegetable	8.38±2.20	11.92±2.87b	8.06±2.44	11.286	<0.001
TMT-A(s)	76.13±19.03	51.75±20.18a	66.88±22.98	5.354	<0.001
SDMT	31.88±12.92	48.63±9.38 b	32.94±10.33	12.000	<0.001
TMT-B(s)	221.88±64.00	151.00±63.75b	225.25±93.80	5.687	0.006
SCWT-C-time(s)	134.00±48.72	82.08±25.84b	119.13±34.61	7.467	0.002
SCWT-C-right	32.13±8.68	44.38±3.66a	34.81±9.38	11.282	<0.001
Similarity test	7.75±1.98	14.00±4.52a	9.00±3.08	11.009	<0.001
CFT-copy	26.63±7.60	34.38±7.26a	26.50±8.33	9.012	0.001
Stick Test -copy	9.00±1.07	9.88±0.45	9.31±0.95	3.102	0.055
Stick Test - rotate	2.00±0.76	5.33±2.68b	2.06±2.52	10.048	<0.001
CDT (Sunderland method)	6.00±1.93	7.79±1.25	6.75±1.34	3.457	0.040

a = *P*<0.01 HE vs. normal group;

b = *P*<0.05 HE vs. normal group; AVLT, Auditory Verbal Learning Test; CFT, Rey-Osterrieth Complex Figure Test; EBPM-VR, event-based prospective memory-verbal response; EBPM-NVR, event-based prospective memory-nonverbal response; TBPM-VR, time-based prospective memory-verbal response; TBPM-NVR, time-based prospective memory-verbal response-non-verbal response; SDMT, Symbol Digit Modalities Test; TMT, Trail Making Test; SCWT, Stroop Color-Word Test; CDT, clock drawing test.

## Discussion

Since the first description of Hashimoto’s encephalopathy (HE) by Brain et al. [27] in 1966, around a hundred cases have been reported worldwide. Most of the published case reports comprise a small sample, and there is no comprehensive statistical analysis of the clinical features associated with HE. In fact, HE remains problematic in terms of its pathophysiology, diagnosis and treatment. The diagnostic criteria proposed by Peschen-Rosin et al. in 1999 [28] encompassed seizures, psychiatric disorders and focal neurological deficits, with elevated thyroid antibodies and an excellent response to steroids. However, this classification of HE underestimates the significance of memory loss and the pathological differences observed in MRI, and overestimates the response to steroids. More and more studies are showing that cognitive impairment is one of the main manifestations of HE, but this aspect has been overlooked due to the multiple and protracted neurocognitive manifestations associated with this condition [27]–[29]. In addition, only 50% of patients with HE are responsive to corticosteroids [29].

The current diagnostic criteria confirm that Hashimoto's encephalopathy is a diagnosis of exclusion [30]. In the current study, patients were diagnosed with HE on the basis of typical clinical manifestations and a high titer of antithyroid antibodies (especially anti-TPO) in serum, and after the exclusion of other causes. We have detected most infectious and immune antibodies finally got the negative results. Otherwise, we carried out the inspection of anti-NMDA receptor antibody and voltage gated potassium channel (VGKC) antibody. A serum screening for these two antibodies was routinely done for differential diagnosis of anti-NMDAR encephalitis and limbic encephalitis, which showed the similar manifestations as HE therefore easily confused with the recognition. In our opinion, patient with anti-NMDAR encephalitis might represent more protracted courses and severe outcomes. The autonomic instability such as hypoventilation and low-grade fever could usually be noted in anti-NMDAR encephalitis, which was scarcely occurred in HE disease. Many anti-NMDAR encephalitis cases were also typically women combined with ovarian teratomas. Our female ones have done routine imaging exam, and found no teratomas. All our patients had negative anti-NMDA receptor antibody and showed better prognosis. We might exclude the anti-NMDAR encephalitis from both clinical manifestation and laboratory examination. Besides, most limbic encephalitis were para-neoplastic disease, since our patients have negative tumor antigens and neuronal nuclear antibodies screening (including anti-Hu IgG, anti-Yo IgG, anti-Ri IgG, anti-CV_2_ IgG et al), and negative serum VGKC antibody as well as the elevated titers of antithyroid antibodies (anti-TPO levels were greater than 1,300 IU/ml in five HE patients; the other three patients had anti-TPO levels greater than 400 IU/ml,Anti-TG levels were greater than 90 IU/ml in all eight cases), thus we might not first consider the diagnosis of the limbic encephalitis.

It is reported that the disease may present in two clinical types, a sudden vasculitic type presented stroke-like episodes or a progressive subacute type associated with cognitive dysfunction, confusion and memory loss [30]. Our study focused on cognitive impairments of the second type; the vasculitic type of HE was scarcely included. Some reports have suggested that there are elevated CSF protein levels in HE. Lumbar punctures were not performed in this study, but future studies should consider including this for laboratory diagnosis.

As there is no gold-standard diagnostic test for HE, neuropsychological tests are an important tool that can help to confirm diagnosis and assess the response to corticosteroid treatment [6]. We carried out a review of international papers that investigated cognitive impairment in HE; the research linking them was limited. Most of the relevant literature concerned case reports, using only Mini-Mental State Examination Scores (MMSE) for a rough assessment [31], [32]. There are few case reports that entail a comprehensive examination of cognitive impairment patterns to estimate the steroid response. Cummings [33] reports that pre-treatment testing revealed global cognitive impairment, with deficits in: overall mental status; simple and complex attention; nonverbal reasoning; line orientation; and list, story and figure learning and recall. In addition, Mazzu [34] and Fukunaga [35] showed a diffuse pattern of cognitive impairment that eventually progressed toward a selective deficit in executive functions and procedural memory. They also suggested that this pattern of cognitive impairment, characterized by widespread brain involvement, primarily implicated the frontal lobe [34], [35].

To investigate the cognitive impairment pattern of HE in a systematic manner, we recruited HE patients with mild, pre-treatment cognitive impairment, and compared the results with mild AD patients, and normal elders. To the best of our knowledge, it is the first statistical analysis of cognitive impairment associated with Hashimoto’s encephalopathy. The results showed that the scores for the HE group were worse than those of the normal control group in most tests. However, there was no difference between these two groups in terms of BNT. This suggested that there was an overall impairment in cognitive function for the HE patients, but long-term semantic memory (naming ability) remained unaffected. This pattern of cognitive impairment was associated with diffuse brain involvement, but rarely implicated the temporal cortex. However, when comparisons were made between the HE group and the mild AD group (who were matched for education, gender and MMSE scores), there was no significant difference in performance, indicating that HE might show a similar pattern of cognitive impairment to AD.

The patients’ cognitive function improved to some extent during or after steroid treatment; MMSE scores were elevated to 28–30/30. This is consistent with the generally accepted viewpoint that treatment with steroids promotes marked clinical improvement and usually infers a good prognosis [27]–[30], [34], [35]. This is of considerable importance for patients with rare but treatable causes of encephalopathy presenting with acute or subacute cognitive decline. Unfortunately, a comprehensive follow-up neuropsychological battery was not carried out. Further studies are needed to verify the detailed pattern of improvement following treatment.

MRI is widely used as a non-invasive means of studying pathological changes in the CNS, and it can provide a unique insight into the pathological status of HE. Among the eight cases of HE in this study, the MRI revealed two types of manifestation associated with HE: the more frequently reported leukoencephalopathy-like type, and limbic encephalitis-like type. Two of the patients (cases 3 and 7) showed the former manifestation-type, with widespread periventricular hyperintense signals in the cerebral white matter (Figure 1(A, B)). Similar pathologies were reported in two other case reports. One was a 65-year-old woman with subacute deterioration of cognitive function, whose brain MRI revealed diffuse high intensity in the white matter on diffusion and T2-weighted images, which mimicked leukoencephalopathy [36]. The other was a 50-year-old man presenting with lower motor neuron symptoms that had evolved over three years, and changes in behavior associated with attentive and cognitive impairment. This patient’s cranial MRI also revealed multiple subcortical white matter lesions [37].

In the current study, limbic encephalitis pathology was observed in cases 2 and 4. The foci of hypersignal on FLAIR for these patients were the bilateral/unilateral medial temporal and neighboring structures (i.e., hippocampus and amygdale; Figure 2(A, B)). Similar findings have been reported previously. Shindo et al. reported an HE case, whose brain FLAIR MRI demonstrated near-symmetrical high signal intensity areas in the bilateral mesial temporal lobes [38]. Lesions in the neighboring structures of the limbic lobe have occasionally been reported, such as localized symmetrical lesions in the bilateral pallidum to the genu of the internal capsule [35]. The remaining four cases in this study showed mild or severe hippocampus swelling but no signal intensity. On the basis of the neurological/psychiatric impairment, we believe that there should be a lesion present in these cases, which may be discovered using functional MRI (fMRI), e.g., blood oxygen level dependent (BOLD) fMRI, or DTI (diffusion tensor imaging). On the whole, the pathology revealed by MRI showed no involvement of the temporal cortex, which is consistent with the cognitive impairment findings that show no deterioration in long-term semantic memory function.

However, it should be noted that the information provided by this article only showed the observed phenomena at a specific time point rather than over a period of time. We are currently performing a longer-term follow-up of patients after treatment to evaluate any residual lesions and treatment efficacy. Therefore, more studies are needed to confirm the conclusions of our study and extrapolate these patients’ results in larger cohorts.

### Conclusions

Our findings suggest that the impairments in cognitive functions for the HE patients were similar to those of AD patients except the remained naming ability. The MRI of HE showed leukoencephalopathy-like or limbic encephalitis-like type; the lesions did not affect the temporal cortex which plays a role in naming ability.
